# Diagnostic Yield of Trio Whole-Genome Sequencing in Children with Undiagnosed Developmental Delay or Congenital Anomaly: A Prospective Cohort Study

**DOI:** 10.3390/diagnostics14151680

**Published:** 2024-08-02

**Authors:** Jaewon Kim, Jaewoong Lee, Myungshin Kim, Dae-Hyun Jang

**Affiliations:** 1 Department of Physical Medicine and Rehabilitation, Incheon St. Mary’s Hospital, College of Medicine, The Catholic University of Korea, Seoul 06591, Republic of Korea; 2 Department of Laboratory Medicine, Incheon St. Mary’s Hospital, College of Medicine, The Catholic University of Korea, Seoul 06591, Republic of Korea; ljw@catholic.ac.kr; 3 Department of Laboratory Medicine, Seoul St. Mary’s Hospital, College of Medicine, The Catholic University of Korea, Seoul 06591, Republic of Korea

**Keywords:** developmental delay, congenital anomalies, whole-genome sequencing, genetic testing

## Abstract

Developmental delays (DD) and congenital anomalies (CA) are prevalent yet often remain undiagnosed despite comprehensive genetic testing. This study aims to investigate the diagnostic yield of trio whole-genome sequencing (WGS) in children presenting with DD or CA who remained undiagnosed after previous genetic testing. A prospective cohort study was conducted on children with undiagnosed DD or CA at a single tertiary hospital. All participants suspected of genetic conditions had undergone chromosome analysis, chromosome microarray analysis (CMA), and clinical exome sequencing (CES); however, a subset remained undiagnosed. The WGS test was administered to both the affected children and their parents. A total of 52 children were included, and 10 (19.2%) had undergone a genetic diagnosis through WGS. Eight of these cases were associated with autosomal dominant and de novo variants. WGS led to successful diagnosis due to several factors, including small structural variants, genes not covered in the CES panel, the discovery of newly implicated genes, issues related to coverage depth, low variant allele frequency, challenges in variant interpretation, and differences in the interpretation of variants of unknown significance among clinicians. This study highlights the clinical value of trio WGS testing in undiagnosed children with DD or CA. Notably, an additional 19.2% of affected children were diagnosed through this method.

## 1. Introduction

Children with developmental delay/congenital anomaly (DD/CA) comprise approximately 3% of the general population [1]. Considerable efforts are being made to identify the genetic causes of these conditions for the purposes of disease understanding and family planning. Prior research has investigated the utility of genetic testing, leading to a global consensus on the clinical usefulness of tests such as chromosomal microarray analysis (CMA) and exome sequencing (ES).

Recent advancements in bioinformatics and genetic technology have identified over 3000 genomic anomalies associated with DD/CA. Notably, advancements in whole-genome sequencing (WGS) have enabled the diagnosis of not only single or short nucleotide variants identified through ES and large structural variants identified through CMA but also noncoding regions, such as introns and regulatory regions, mitochondrial genomes, short tandem DNA repeat expansion disorders, and copy-number variants of various sizes (under 5–10 kb) [2]. The initial issues of low depth in early WGS have been largely resolved, enhancing its accuracy. Recent developments have progressed to the extent that Sanger sequencing may no longer be necessary for sequencing validation, demonstrating significant advancements in precision [3,4].

Previous studies have shown that the overall diagnostic yield of genetic tests for neurodevelopmental disorders ranges between 21% and 41%, whereas for WGS, it is around 43%. It is anticipated that these diagnostic yields will increase in the future. Consequently, it has been suggested to employ clinical-level exome sequencing (CES), whole-exome sequencing (WES), or WGS as a primary or secondary test [5,6,7]. Trio testing, in particular, has been demonstrated to have a higher diagnostic yield than testing the proband alone and is especially crucial in diagnosing rare diseases [8]. The present study aimed to report the diagnostic yield of the WGS trio test in children with DD/CA who were suspected of having genetic abnormalities but remained undiagnosed after chromosome analysis, CMA, and CES. Furthermore, this study analyzed the reasons for the failure of diagnosis using previous genetic tests.

## 2. Materials and Methods

### 2.1. Ethical Considerations and Patient Consents

The children enrolled in this study and their parents voluntarily agreed to participate, and written informed consent was obtained from all parents before collecting blood samples. The Institutional Review Board approved this study, confirming its adherence to relevant guidelines and regulations (ethics approval number: KC20TNDI0552). This research was conducted under the Korean National Project of BioBig Data (No. 2020M3E5D7086780).

### 2.2. Study Participants and Design

In this prospective cohort study, children with DD or CA aged < 18 years who were suspected of having genetic abnormalities but remained undiagnosed after chromosome analysis, CMA, and CES were recruited. Trio WGS was performed at our clinic and those aged > 18 years who were under continuous observation since early childhood (under 6 years) were included. Children with DD who only exhibited language DD were excluded. Children with DD were categorized into those with intellectual disability (ID) and those with gross motor delay. Children with ID were included if they demonstrated a significant decline in intellectual and adaptive function, as assessed by developmental evaluations (such as the Wechsler Intelligence Scale and Bayley Scales of Infant and Toddler Development) conducted after 36 months of age. Children with gross motor disorders were included if they exhibited DD in gross motor skills or demonstrated disorders of muscle tone or movement. Patients with CA were included if two or more major anomalies were confirmed. The inclusion criteria for this study were as follows: (1) children diagnosed with DD (including ID or gross motor disorder) or CA; (2) absence of prenatal or perinatal issues, exposure to exogenous factors such as alcohol or drugs during pregnancy, or infections that could lead to DD/CA; (3) children with no other abnormalities detected on performing specific genetic tests (e.g., triplet repeat primed PCR and methylation-specific PCR restriction fragment length polymorphism); and (4) children with no abnormalities detected on chromosome analysis, CMA, and CES. Detailed methodologies for chromosome analysis, CMA, and CES have been described in a previous study [9]. Clinical exome sequencing used in this study is an NGS test that utilizes an exome panel containing more than 4800 genes so that it can be used for clinical purposes. CES was performed solo, but pathogenic variants suspected to be de novo were verified by Sanger sequencing in trio. The exclusion criteria included cases with non-genetic or acquired causes of DD/CA and children with suspected DD who did not manifest clear ID or gross motor delay.

**Figure 1:**
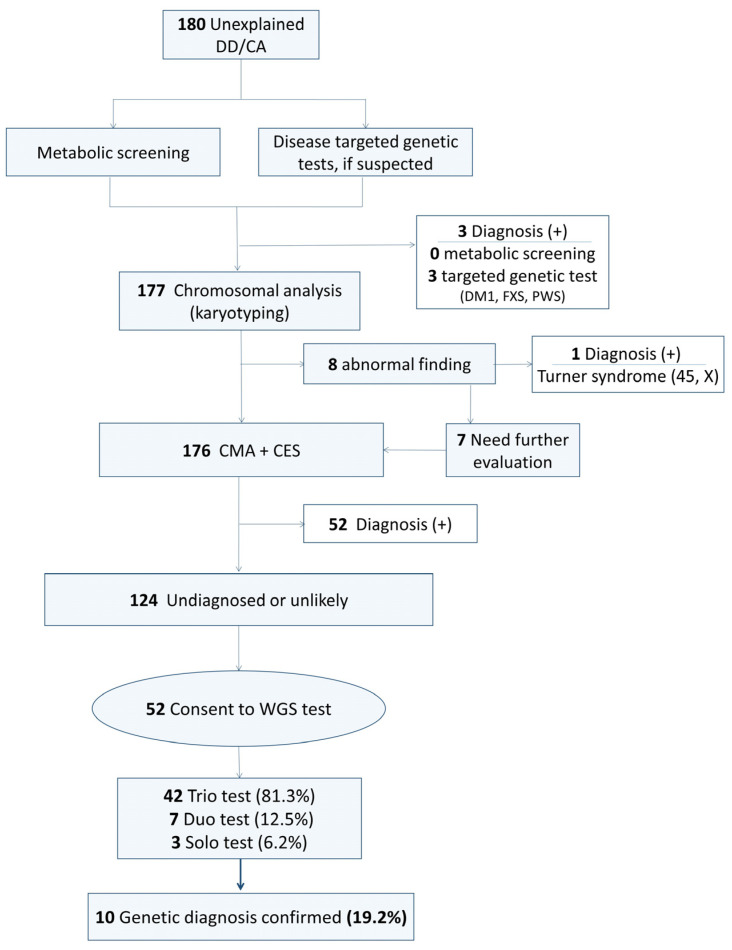
Study flow diagram. DD, developmental delay; CA, congenital anomaly; DM1, myotonic dystrophy type 1; FXS, Fragile X syndrome; PWS, Prader-Willi syndrome; CMA, chromosomal microarray analysis; CES, clinical exome sequencing; WGS, whole-genome sequencing.

Consequently, for cases identified as having unexplained DD or CA, WGS was conducted. Trio WGS testing was recommended wherever possible; however, duo or solo WGS testing was performed in situations where trio testing was not feasible (due to circumstances such as parental divorce, bereavement, or refusal to participate in the test). If the proband’s sibling exhibited a similar phenotype, sibling testing was also conducted. The flow chart of the study is illustrated in Figure 1. For the subjects of the study, data were collected from previous tests, including chromosome analysis, CMA, CES, and clinical data such as combined phenotypes and brain MRI results.

### 2.3. WGS and Data Analysis

Genomic DNA was extracted from peripheral blood, and the purified DNA was fragmented into short sequences. WGS was performed using the novaseq6000 platform (Illumina, CA, USA). Bioinformatic analysis was performed using Burrows-Wheeler Aligner v.2.2.1 and samtools v.1.12, including alignment of fastq data into Genome Reference Consortium Human Build 38 (GRCh38). Single nucleotide variants (SNVs) and small insertion/deletion (indel) variants were identified with GATK v.4.2, while structural variants (SVs) were identified with MANTA v.1.6.0 and cn.MOPS v.1.38.0. For variant annotation, Ensemble Variant Effect Predictor v.104 was used for SNV/small indel variants and AnnotSV v.3.1 for SVs. All variant annotations were based on GRCh38. The annotated variants were classified according to the 2015 American College of Medical Genetics and Genomics and the Association for Molecular Pathology (ACMG/AMP) guidelines for pathogenicity [10]. The bioinformatics pipeline did not include a section to check for the presence of mtDNA mutations, balanced chromosomal translocations, and FMR1 expansion, but chromosome testing and FMR1 expansion were confirmed for each patient through separate tests.

## 3. Results

### 3.1. Clinical Characteristics of Patients

The study included a total of 52 children (33 males and 19 females), with an average age of 6.5 years. Among them, 51 had DD, including 42 with ID and 25 with gross motor disorder. Twelve children presented with CA, and seven children exhibited a combination of ID, gross motor delay, and CA. Figure S1 illustrates the classification and count of participating children. Of the included patients, 41 (78.8%) underwent trio WGS (proband + parents), 7 (13.5%) underwent duo (proband + one parent), 3 (5.8%) underwent solo (proband only), and 1 (1.9%) underwent penta (proband + both parents + two other affected siblings). The interval between CMA and CES testing and the WGS analysis was an average of 6.3 months (SD 11.7 months, range 2–23 months). Detailed clinical information about the children included in the study is provided in Table 1.

### 3.2. Diagnostic Yield of WGS after Chromosome Analysis, CES, and CMA

Out of the 52 children who received negative results from chromosome analysis, CES, and CMA, 10 (19.2%) were diagnosed via WGS. Detailed information about these 10 probands is provided in Table 2. Among the 51 children with DD, 9 (17.6%) were diagnosed [7 out of 42 children with ID (16.7%), and 8 out of 25 children with gross motor delay (32.0%)]. Additionally, 3 out of 12 children with CA (25%) were diagnosed. Among the children who received a final genetic diagnosis through WGS, 8 underwent trio testing, while one child each underwent duo and solo testing.

### 3.3. Analysis of the Variants Identified through WGS

Among the 10 probands in whom causal genes for ID, gross motor delay, or CA were identified through WGS, 8 were de novo and autosomal dominant (80%), and 2 were autosomal recessive (AR) inherited from both parents (20%). This led to the identification of a total of 12 variants in these 10 probands. According to the ACMG classification, 11 of these 12 variants (91.7%) were pathogenic and 1 (8.3%) was likely pathogenic (LP). The variant classified as LP was NT5C2 c.1106T>C. This gene is associated with spastic paraplegia 45 through AR inheritance. It was inherited from the proband’s mother, and in conjunction with the pathogenic variant inherited from the proband’s father (NT5C2 c.1159+2T>G), it resulted in the manifestation of spastic paraplegia 45.

### 3.4. Factors Contributing to Non-Diagnosis by Chromosome Analysis, CMA, and CES

The causes for non-diagnosis by chromosome analysis, CMA, and CES could be categorized into several types. These include (1) small structural variants, (2) genes not encompassed in the CES panel, (3) discovery of newly implicated genes, (4) issues related to depth of coverage, (5) low variant allele frequency (VAF), (6) challenges in variant interpretation (where previously reported phenotypes differed from the proband’s clinical presentation), and (7) interpretation differences of variants of unknown significance (VUS) among clinicians.

Patient 1 had a large indel variant that could not be identified through conventional NGS methods. A 3.3 kb indel variant in ASXL3 was detected using the structural variant calling pipeline in WGS. This variant was not identifiable by CES as it involves a deep intronic region not covered by the CES analysis pipeline. Variants in genes not included in the CES panel were identified in patients 2, 3, 4, and 8.

**Figure 2:**
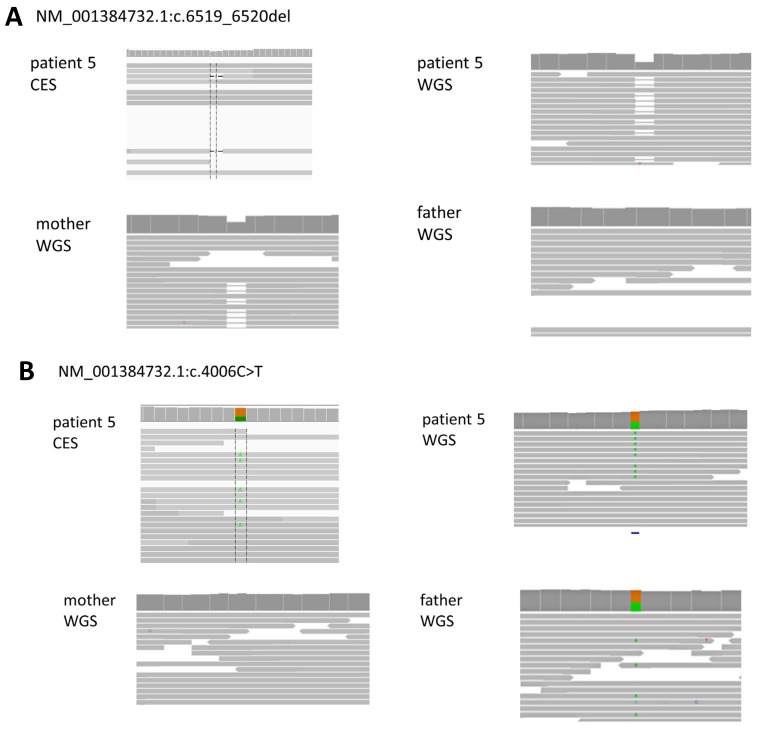
Read status of *CPLANE1* pathogenic variants in patient 5 in CES and WGS. (**A**) The deletion variant (NM_001384732.1:c.6519_6520del), which was not detected in CES, exhibited only 2 variant reads, whereas in WGS, it had 9X variant reads. (**B**) The missense variant (NM_001384732.1:c.4006C>T) showed sufficient variant read depth in both CES and WGS.

In the case of patient 5, two different variants of the same gene were identified as compound heterozygous in an AR disease. However, one of these variants was not identified in CES due to a lack of read depth. This could be attributed to factors such as an insufficient total read count, the characteristics of the hybrid capture probe, or low uniformity. After conducting quality control checks, it was determined that the total read count was adequate and the uniformity was above 95%. Nevertheless, the locus that was not identified exhibited a significantly lower total read depth, likely due to the nature of the hybrid capture probe used in the test (NM_001384732.1:c.6519_6520del: CES total read depth = 9, WGS total read depth = 33). This was confirmed with the Integrative Genomics Viewer, and the read status of the variant region is depicted in Figure 2.

Patients 6 and 7 exhibited high total read depths for the observed variant loci but were excluded from the CES analysis due to their low VAFs of 11.2% and 6.3%, respectively. In our laboratory’s routine CES analysis, VAFs below 20% are typically filtered out during interpretation. The VAFs in WGS were 19% and 32%, respectively. Given that the VAFs of these variants were low in both CES and WGS, the possibility of mosaicism cannot be dismissed. These variants are not observed in other samples tested together and are observed with equally low VAF across different kits and bioinformatic pipelines, so they can be considered not artifacts. Although there was no opportunity for further analysis, verification by Sanger sequencing would have been helpful in confirming mosaicism.

In the case of patient 8, a pathogenic variant in H1–4 was identified. This suggests that this gene, which has recently been recognized as a cause of DD/ID, was not included in the CES panel.

In the cases of patients 9 and 10, the variants were also identified in the CES readings but were not deemed causative for the patient phenotypes during interpretation. The clinical presentation of patient 9 did not encompass microphthalmia, which is typically associated with SOX2-related diseases. However, rare cases of patients with SOX2-related diseases but without microphthalmia have been reported, underscoring the need for careful consideration by the interpreter. For patient 10, the c.1106>C variant in NT5C2 was classified as a VUS in the CES reading due to its high frequency in the East Asian population (0.00003), leading to an inappropriate application of the BS2 criteria of the ACMG/AMP guidelines [10]. However, since the related disease is AR, an LP classification would be more appropriate without the erroneous application of BS2.

### 3.5. Genetic Diagnosis and Impact on Medical Care (Treatment Impact)

Patient 9, born at 35 weeks and weighing 2170 g, was diagnosed with cerebral palsy due to persistent gross motor developmental delays since infancy. The patient received several botulinum-toxin injections and underwent physical and occupational therapy for spasticity. By 48 months, the patient could walk independently, with noticeable improvements in the afternoon compared to mornings, and exhibited dystonic and dyskinetic movements. At 9 years old, the Wechsler Intelligence Scale for Children yielded a full-scale IQ of 35, indicating ID. At 12 years old, the trio WGS test conducted as part of this study identified a de novo SOX2 c.310G>T (p.Glu104Ter), a novel variant. SOX2-related disorders are known to include DD, ID, ophthalmological issues like microphthalmia, brain malformations, hypogonadism, and gonadal dysgenesis. The patient did not exhibit any clinical symptoms, but an ophthalmological examination revealed myopia and a retinal disorder. The condition is currently being monitored with eyeglass treatment. Pelvic MRI conducted for gynecological examination revealed a hypoplastic uterus and ovarian agenesis, and hormone therapy is currently being considered.

### 3.6. Some Case Presentations on Variants Identified through WGS

Patient 1, who was previously diagnosed with cerebral palsy and exhibited severe ID and gross motor function impairment, had developmental levels evaluated as below 12 months at the age of 18 years. A trio WGS revealed a small structural variant in ASXL3 (NC_000018.10: g.33740509_33743845delinsAGAAGCCTAGGTGTAC), measuring 3.3 kb. This variant underscores the utility of WGS in identifying deep intronic variants and small SVs that are challenging to detect via CMA.

Patient 3, with normal development, presented with unilateral scapular winging and laterocollis. CT scans revealed cervical spine fusion (C2–3), spina bifida (C3, 4, 6), and Sprengel’s deformity (See Figure S2). Additional tests, including a whole-body X-ray, echocardiography, and abdominal ultrasound, did not reveal any remarkable findings apart from an incidental 5 × 7-mm small urachal cyst. This case suggests the potential for genetic diagnosis through WGS even in conditions with single-organ skeletal deformities.

## 4. Discussion

This study assessed the diagnostic effectiveness of trio WGS tests in children with DD/CA who remained undiagnosed after comprehensive genetic tests, including chromosome analysis, CMA, and CES. Out of 52 children, 10 (19.2%) were diagnosed through WGS, with 8 cases (80%) being autosomal dominant and de novo. The reasons for nondiagnosis through previous genetic tests varied, including a small SV, variants in genes not included in the CES panel, a newly identified gene, insufficient depth, low VAF, and issues in variant interpretation. 

In this study, 7 out of 42 children with ID were diagnosed through WGS, i.e., the diagnostic yield was 16.7%. Our team previously found that the combined diagnostic yield of CMA and CES in 154 children with ID was 25.9–33.7%, with CMA and CES accounting for 12.3–14.3% and 13.6–19.4% of the yield, respectively [9]. Of the 102 children with ID who remained undiagnosed, 30 underwent WGS, leading to 4 additional diagnoses and a diagnostic yield osf 13.3%. All variants detected through CMA and CES could be detected through WGS, suggesting a potential diagnostic yield of up to 47% when trio WGS is used as the primary test in children with unexplained ID. A previous study reported a 21% increase in diagnoses in children with ID who had not been diagnosed through conventional genetic tests such as CMA or WES when subsequently tested with WGS [11]. However, since these children did not undergo both tests before WGS, the diagnostic yield reported in that study might have been higher than that reported in the present study [11]. Previous studies have predicted the diagnostic yield of WGS in neurodevelopmental disorders to be up to 41% [7,12]. Our results demonstrate an estimated diagnostic yield of over 39%, which is comparable to that reported in a previous study [9].

Recent technological advancements have highlighted cases where abnormalities in the noncoding regulatory region disrupt gene regulation, leading to neurodevelopmental disorders due to alterations in brain development and function [13,14]. With the progression of WGS, especially in identifying pathogenic variants in noncoding regions, the diagnostic yield is expected to increase further. Furthermore, the accumulation of genetic data from newly discovered genes or novel variants through WGS research is anticipated to further enhance diagnostic rates.

In the case of patient 5, the difference in read depth between CES and WGS was crucial for variant detection. This difference is inherent in the testing method itself. CES, being a large targeted panel sequencing, uses a hybrid capture method that inevitably results in regions of low read depth depending on the capture probe’s position. Consequently, this leads to lower sensitivity compared to WGS, even within the coverage area of CES.

In this study, one child was identified with a newly recognized gene reported in recent research as disease-causing. Two other children had genes that were not included in the enrichment kit. Therefore, out of the 10 children, a total of 4 might have been diagnosed with panel updates and reanalysis. In settings where WGS is challenging and only CES is feasible, periodic reanalysis could enhance diagnosis while considering cost-effectiveness [15]. Reanalysis is also advantageous for WGS, given the increasing identification of more genes with its expanding use. Indeed, reanalysis of next-generation sequencing data in patients with neurodevelopmental and rare diseases has resulted in new diagnoses in approximately 18%–53% of patients, particularly when conducted after 24 months [16,17]. Reanalysis of WES data over three years has led to additional genetic diagnoses in over 15% of cases [18]. This underscores the significance of periodic reanalysis in sequencing-based gene studies, and we intend to reanalyze the subjects from this study.

In this study, two cases remained undiagnosed due to interpretation issues. Patient 9 presented an unreported phenotype, while patient 10 exhibited spasticity and gross motor delay, along with an incidental brain anomaly, specifically corpus callosum hypoplasia. To minimize such interpretation errors, communication between clinicians and geneticists is crucial. However, considering that variants not detected due to low depth in CES cannot be identified even in reanalysis unless a high throughput environment used in GS is employed, it is reasonable to consider that excluding the two cases related to variant interpretation, the remaining eight cases could have been diagnosed solely with GS. Of course, even in the case of GS, sufficient depth is the basic requirement for variant detection.

A key strength of this study was that it focused on children who remained undiagnosed after previous tests, enabling the evaluation of the diagnostic yield of WGS and highlighting the significant potential of WGS in identifying elusive diagnoses where standard genetic testing methods, such as CMA and CES, fall short. Notably, most CMA and CES tests were conducted after 2021. Furthermore, the use of trio WGS was instrumental in identifying the cause of VUS and LP de novo variants and in confirming the inheritance in AR diseases, thereby enhancing the diagnostic yield.

The limitations of this study include the challenge of statistical analysis due to the small sample size. However, given the nature of rare diseases, the study’s results hold value irrespective of statistical analysis. While trio testing is beneficial for detecting de novo variants in dominant genes from unaffected parents, it can pose challenges in cases of mosaicism originating from unaffected parents. This was considered during the analysis, and efforts were made to thoroughly consider clinical phenotypes in our analysis. Also, although all patients were equally informed, a total of 52 families out of 124 consented to WGS, and there may have been a selection bias, such as families with children showing more severe symptoms being more likely to consent. Additionally, time and distance constraints likely influenced participation, and this is acknowledged as a limitation of the study.

## 5. Conclusions

Trio WGS enabled additional genetic diagnoses in 19.2% of children with unexplained DD or CA, even when chromosome analysis, CMA, and CES tests yielded negative results. The successful diagnoses through WGS were attributed to various factors, including the identification of small SVs, genes not included in the CES panel, the discovery of newly implicated genes, insufficient depth, low VAF, and issues in variant interpretation.

## Figures and Tables

**Table 1:** Clinical characteristics of study subjects.

	N (Total = 52)	Whole Genome Sequencing	
		Diagnosis (+)	Diagnosis (−)
Age (mean), yr	6.5		
Sex			
Male	33	6	27
Female	19	4	15
Developmental delay	51		
Intellectual disability	42	7	35
Gross motor delay	25	8	17
Congenital anomaly	12	3	9
Brain MRI abnormality (N = 48)	8	3	5
Combined phenotype *			
Skeletal	10	2	8
Ophthalmology	7	3	4
Gastrointestinal	3	2	1
Cardiac	2	1	1
Epilepsy	2	0	2
Gynecology	1	1	0

* An individual may have multiple phenotypes.

**Table 2:** Variants identified through whole-genome sequencing.

Patient No.	Sex/Age	Genotype	Inheritance (Origin)	Disorder	Phenotypes	Reasons for Non-Detection in Previous Tests *
1	F/18	*ASXL3 * NC_000018.10: g.33740509_33743845delins AGAAGCCTAGGTGTAC, 3.3 kb	AD (De novo)	Bainbridge-Ropers syndrome	ID, gross motor delay, hypotonia, speech delay, dysmorphic face	Small structural variant
2	M/3	*SPEN* chr1:g.15928344C>T, NM_015001.3:c.2104C>T, (p.Arg702Ter)	AD (De novo)	Radio-Tartaglia syndrome	Gross motor delay, pes cavus, 4th toe underlapping	Identified in genes not included in the CES panel
3	M/4	*WBP11* chr12:g.14787469G>GC, NM_016312.3:c.1521dup, (p.Arg508AlafsTer41)	AD (De novo)	VCTERL (Vertebral, cardiac, tracheoesophageal, renal, and limb defects) syndrome	Cervical spine fusion (C2–C3), spina bifida (C3, C4, C6), Sprengel deformity, urachal cyst	
4	M/2	*GRIN2B* chr12:g.13571953A> AGGTCTCTGGAACT, NM_000834.5:c.2011-2_2021dup, (p.ASn675ValfsTer5)	AD (De novo)	GRIN2B-Related Neurodevelopmental Disorder	ID, gross motor delay, hypotonia, ptosis, microcephaly (Brain MRI) mild brain atrophy, hypomyelination	
5	F/27	*CPLANE1* chr5:g.37187488G>A NM_001384732.1:c.4006C>T, (p.Arg1336Trp)pat *CPLANE1* chr5:g.37169503CCT>C, NM_001384732.1:c.6519_6520del, (p.Gly2174ThrfsTer37)mat	AR (Parental)	Joubert syndrome 17	ID, gross motor delay, ataxia, dizziness, disconnected speech, severe articulation disorder (Brain MRI) cerebellum (superior vermis) hypoplasia	Insufficient depth
6	M/3	*TRIP12* chr2:g.229771526G>A, NM_001348323.3:c.5801C>T, (p.Pro1934Leu)	AD (unknown)	Clark-Baraitser syndrome (Intellectual developmental disorder, autosomal dominant 49)	ID, autism spectrum disorder	Low VAF (11.2%, 11/98 depth)
7	M/3	*MEIS2* chr15:g.36950363GCTAA>G, NM_170675.5:c.934_937del, (p.Leu312ArgfsTer11)	AD (De novo)	Cleft palate, cardiac defects, and impaired intellectual development	ID, gross motor delay, hypotonia, speech delay, dysmorphic face, VSD, scoliosis, wide 1–2 toe web	Low VAF (6.3%, 4/64 depth)
8	M/12	*H1-4* chr6:g.26156798G>GA, NM_005321.3:c.410dup, (p.Pro138AlafsTer58)	AD (De novo)	Rahmman syndrome	ID, gross motor delay, hypotonia, congenital megacolon	Newly identified gene
9	F/12	*SOX2* chr3:g.181712670G>T, NM_003106.4:c.310G>T, (p.Glu104Ter)	AD (De novo)	SOX2 Disorder	ID, gross motor delay, dyskinesia, spasticity, myopia, retinal disorder, hypoplastic uterus, ovarian agenesis	Variant interpretation issues (reported phenotype differs from proband’s clinical symptoms)
10	F/4	*NT5C2* chr10:g.103093137A>C, NM_001351169.2:c.1159+2T>G p.? pat *NT5C2* chr:10:g.103093192A>G, NM_001351169.2:c.1106T>C, (p.Phe369Ser)mat	AR (Parental)	Spastic paraplegia 45	Gross motor delay, spasticity (Brain MRI) corpus callosum hypoplasia	Differing interpretations of VUS among clinicians

AD, autosomal dominant; AR, autosomal recessive; ID, intellectual disability; VSD, ventricular septal defect; CES, clinical exome sequencing; VAF, variant allele frequency; VUS, variant of uncaptain significance. * Previous tests included chromosome analysis, chromosomal microarray analysis (CMA), and CES. Patients who showed negative results in these tests underwent whole genome sequencing testing.

## Data Availability

The data that support the findings of this study are available from the corresponding author upon reasonable request.

## Supplementary Materials

The following supporting information can be downloaded at: https://www.mdpi.com/article/10.3390/diagnostics14151680/s1, Figure S1: Classification and number of participating children; Figure S2: A 4-year-old boy with a pathogenic variant in *WBP11* (c.1521dup). (A) Sprengel deformity is characterized by an elevated right scapula without connection to the vertebrae and ribs and spina bifida in C3, C4, and C6 vertebrae. (B) Fusion of the right facet joints of C2 and C3, and a large sesamoid ossicle of the nuchal ligament at C3 to C5 level.
