# Supplementary figures and images for: Diagnostic Yield of Trio Whole-Genome Sequencing in Children with Undiagnosed Developmental Delay or Congenital Anomaly: A Prospective Cohort Study
Source: Diagnostics (Basel). 2024 Aug 2;14(15):1680. doi: 10.3390/diagnostics14151680 (PMC11312062; doi:10.3390/diagnostics14151680)

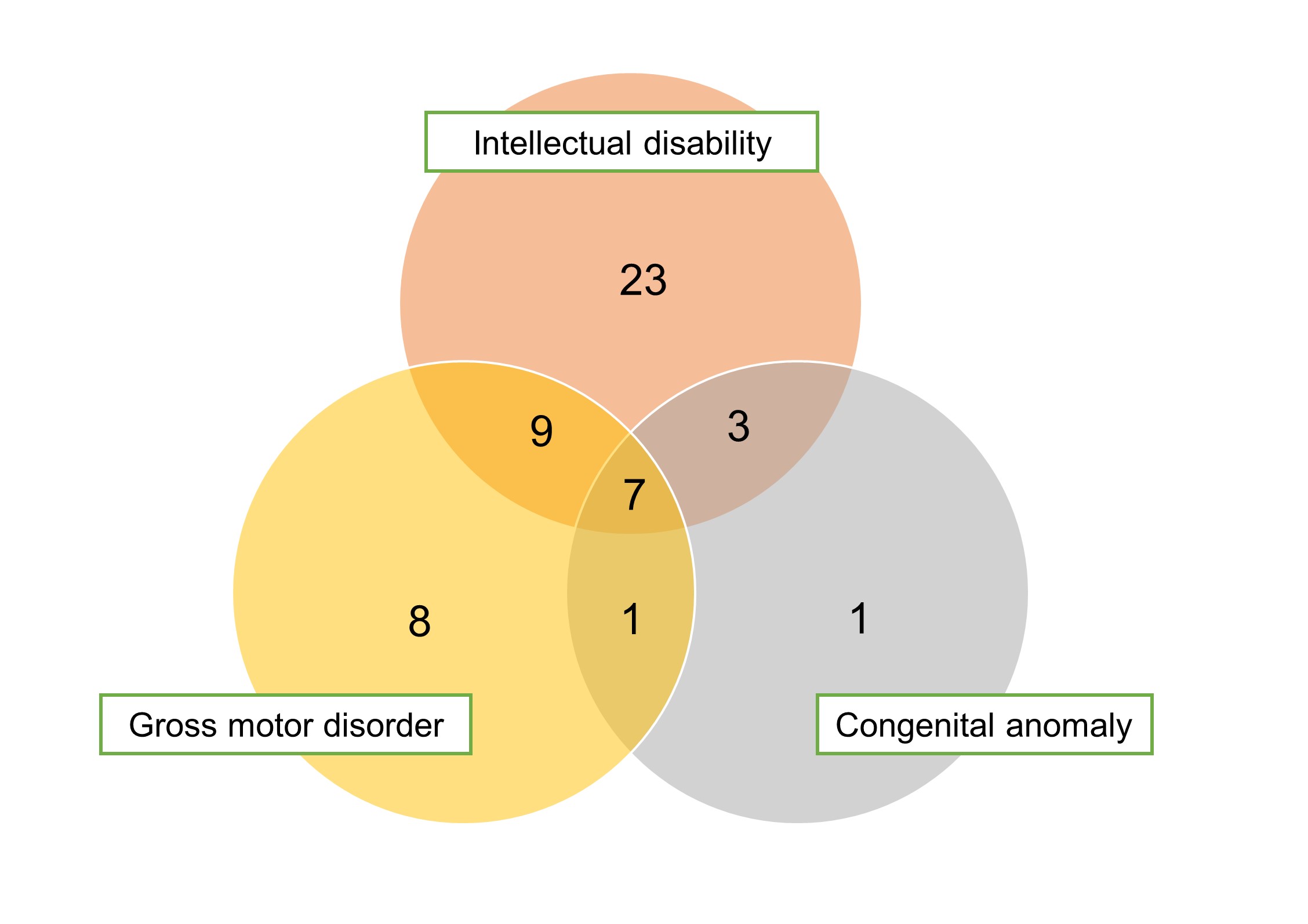

Supplement: Supplementary file 1 [file diagnostics-14-01680-s001.zip › figure S1.jpg]

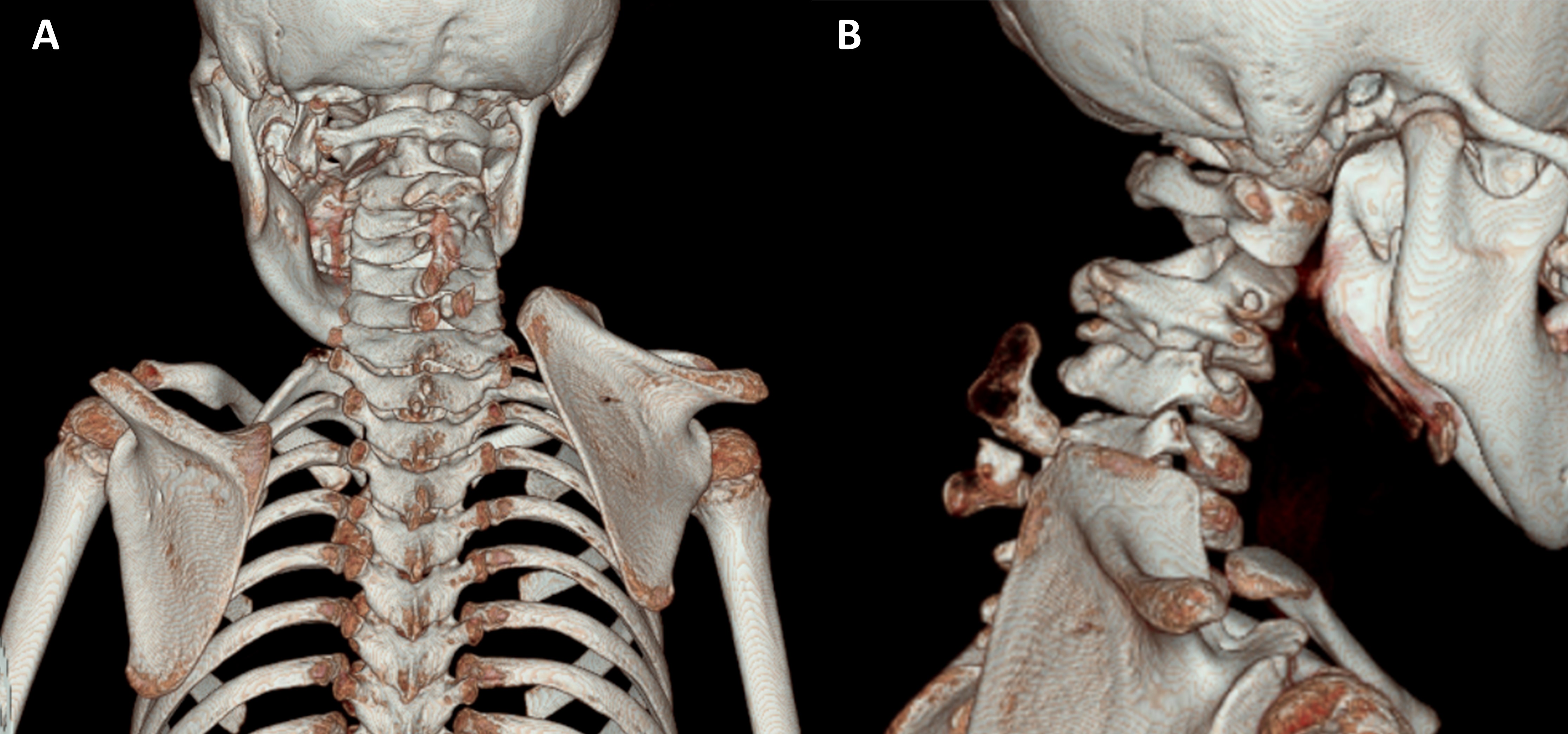

Supplement: Supplementary file 1 [file diagnostics-14-01680-s001.zip › figure S2.jpg]
